# Immunometabolic Changes Following Gastric Bypass and Sleeve Gastrectomy: A Comparative Study

**DOI:** 10.1007/s11695-024-07598-3

**Published:** 2025-01-11

**Authors:** Tania Rivera-Carranza, Alejandro Azaola-Espinosa, Rafael Bojalil-Parra, Eduardo Zúñiga-León, Angélica León-Téllez-Girón, Martín E. Rojano-Rodríguez, Oralia Nájera-Medina

**Affiliations:** 1 Departamento de Atención a la Salud, División de Ciencias Biológicas y de la Salud, Universidad Autónoma Metropolitana, unidad Xochimilco, Coyoacán, Ciudad de México, 04960 Mexico; 2 Departamento de Sistemas Biológicos, División de Ciencias Biológicas y de la Salud, Universidad Autónoma Metropolitana unidad Xochimilco, Coyoacán, Ciudad de México, 04960 Mexico; 3 Academia de Nutrición y Salud, Colegio de Ciencias y Humanidades, Universidad Autónoma de la Ciudad de México plantel Casa Libertad, Iztapalapa, Ciudad de México, 09620 Mexico; 4 Clínica de obesidad, Hospital General Dr. Manuel GEA González, Tlalpan, Ciudad de México, 14080 México

**Keywords:** Bariatric surgery, B cells, Effector T cells, Cytotoxic T cells, Memory T cells

## Abstract

**Background:**

Immunometabolism is the interaction between immune system and nutrient metabolism. Severe obesity is considered a state of meta-inflammation associated with obesity that influences the development of chronic-degenerative diseases.

**Objective:**

We aimed to establish the immunometabolic differences in bariatric patients and to determine whether cellular immunity is associated with metabolic changes.

**Methodology:**

We conducted an observational study in patients who underwent laparoscopic sleeve gastrectomy (LSG) or laparoscopic Roux-en-Y gastric bypass (LRYGB). We explored the differences in the immunometabolic profile before and after surgery in the study group, by surgical technique, and we evaluated the changes in immunological variables as a function of metabolic variables with correlation analysis.

**Results:**

The follow-up rate was 88.7%. After the intervention, there were changes in cellular immunity, with a decrease in effector T lymphocytes (CD8+CD28−) and an increase in B lymphocytes, memory helper T cells, and cytotoxic T lymphocytes. LSG resulted in a greater decrease in (CD4+CD62−) T lymphocytes compared with LRYGB. Patients who underwent surgery with LRYGB presented greater clinical and metabolic improvements, as well as improvement of obesity-associated medical problems. Women who underwent LRYGB showed a greater reduction in fat-free mass compared with women who underwent LSG.

**Conclusion:**

Bariatric surgery, mainly LRYGB, leads to immunometabolic changes and improves associated medical problems.

**Graphical Abstract:**

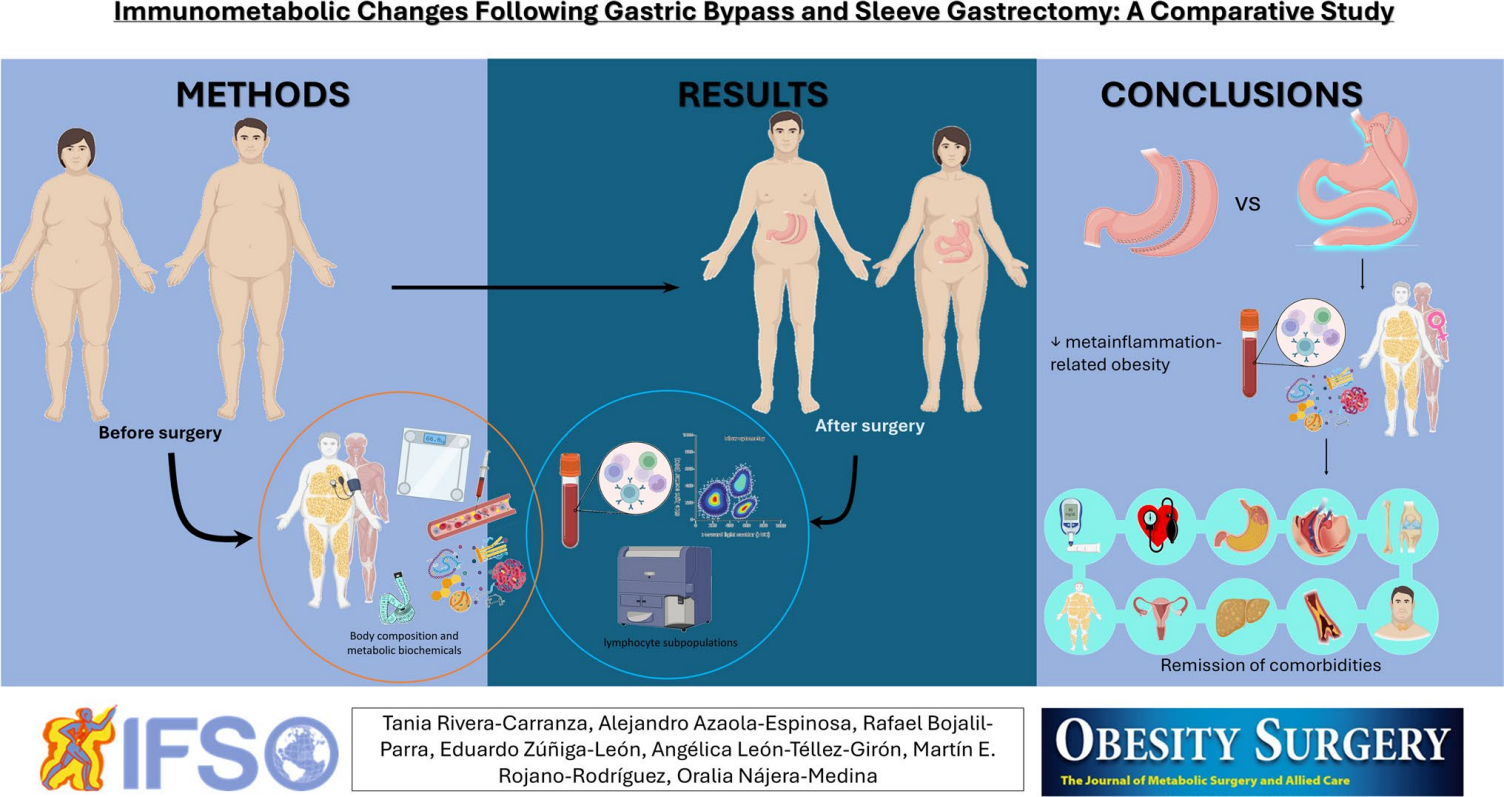

## Introduction

Immunometabolism is the interaction between the immune system and nutrient metabolism [1]. It also refers to how obesity or metabolic/bariatric surgery (MBS) affects the function and metabolic pathways of immune cells and vice versa [2, 3]. Adipose tissue (AT) is an endocrine organ that contains several types of cells, including preadipocytes, adipocytes, fibroblasts, vascular endothelial cells, and immune cells [4, 5]. An ideal immunometabolic profile is observed when adipose tissue does not activate immune cells to maintain inflammation [6]. This occurs in people with a normal total body fat percentage (less than 25% and 30% in men and women, respectively) [7, 8]. In the AT of individuals with obesity, there is hypertrophy and hyperplasia of adipocytes, which lead to the release of fatty acids and, consequently, the activation of innate and adaptive immune cells, generating various immunological stimuli that stimulate the secretion of pro-inflammatory cytokines. Therefore, obesity is considered a state of chronic low-grade inflammation, also known as meta-inflammation-related obesity (referred to as chronic low-grade inflammation state caused by the alteration of the immunometabolism that generates associated medical problems) [6, 7], which leads to the development of chronic-degenerative diseases [8, 9].

Immune cells (leukocytes) divide into different populations and differentiate according to the expression of cluster differentiation (CD) on their membrane and therefore have diverse immune functions. For example, T lymphocytes (CD3+) are divided into (1) helper T lymphocytes (CD4+) that recognize antigens (foreign or toxic molecules for the body). Among them are effector helper T lymphocytes (CD4+CD62−) that activate other immune cells through the secretion of cytokines (a group of proteins that function as means of communication between cells and that directly influence inflammation) and (2) effector cytotoxic T lymphocytes (CD8+CD28−) that directly eliminate affected cells through the secretion of proteases. Both helper and cytotoxic T lymphocytes, in addition to expressing their corresponding CDs, also express (CD45RA + , ship: which gives them the ability to detect new unknown antigens) or (CD45RO+ , memory: ability to generate immunological memory or CD45RA+CD45RO+), T helper, or virgin cytotoxic lymphocytes that are having contact with their specific antigen to become memory cells. B lymphocytes (CD19+) produce antibodies and mark antigens or damaged cells for elimination. NK cells (CD16+CD56+) destroy infected or tumorous cells. Granulocytes (neutrophils, eosinophils, and basophils) mainly destroy bacteria, fungi, or parasites. Monocytes differentiate into macrophages to phagocytose bacteria, necrotic material, or other foreign particles [10].

Individuals with obesity have changes in the proportion and function of their immune cells in AT and peripheral blood. These variations are accentuated as *BMI* and/or total body fat (TBF) increase [6]. There is an increase in the percentage of cellular subpopulations in AT and peripheral blood, including CD4 + T lymphocytes, CD4+CD62− T lymphocytes, and CD4+CD45RO+ T lymphocytes [11–13], as well as senescent T lymphocytes [14]. There is also an increase in B lymphocytes [15–17] and activated and memory cytotoxic T lymphocytes [18–20]. These cells are positively associated with the presence of systemic and metabolic inflammatory markers such as C-reactive protein (CRP) [12], low-density lipoprotein cholesterol (LDL-c) [21–23], and age [24, 25]. Additionally, the natural killer (NK) cells in the blood of individuals with obesity have lower activity, which has been linked to an increased risk of cancer [26–29].

The treatment of obesity involves modifying lifestyle through a plan that includes nutrition, exercise, and behavioral management. In some cases, weight-loss medications may be effective; however, bariatric surgery such as laparoscopic Roux-en-Y gastric bypass (LRYGB) and laparoscopic sleeve gastrectomy (LSG) represents an additional treatment option when previous approaches have failed [30]. After bariatric surgery, there is a reduction in inflammatory mediators, which contributes to improve the immunometabolic profile and, therefore, reduces the risk of morbidity and mortality [31, 32].

The aims of this study were (1) to describe the immunometabolic changes in patients with obesity undergoing bariatric surgery, (2) to determine the association between lymphocyte subpopulations and metabolic improvements, and (3) to investigate which surgical procedure is most favorable for optimizing immunometabolism.

## Materials and Methods

An observational, analytical, and longitudinal study was conducted. It included 50 adults with obesity of both sexes who underwent LRYGB and LSG from 2020 to 2023. The inclusion criteria were patients with obesity who were enrolled for the bariatric surgery protocol in the obesity clinic of the Dr. Manuel Gea Gonzalez Hospital in Mexico City, with or without associated medical problems, and who provided signed informed consent letter. The exclusion criteria were patients with infection, autoimmune diseases, renal disease and/or cancer, or those receiving anti-inflammatory or immunosuppressant drugs. The elimination criteria were individuals who withdrew consent from the study, those with incomplete data, or those with any of the diseases and/or treatments mentioned under the exclusion criteria or who developed a post-surgical complication. The population of patients eligible for surgery was evaluated 1 month before and 6 months after the surgery.

## Anthropometric and Body Composition Measurements and Biochemical and Arterial Blood Tests

Weight and height were measured with a Seca 704 s TM scale with a stadiometer (Seca). The waist circumference (WC) was determined with an Executive 6FT W606P stainless-steel metric tape measure (Lufkin W606PD Executive Thinline). These were carried out following the standardized protocol of the International Society for the Advancement of Kinanthropometry (ISAK). The BMI, TBF, free fat mass (FFM), and visceral fat (VF) were obtained using a BC-568 body composition analyzer (Tanita). The equation used to calculate excess weight lost (EWL) = (weight lost) × 100/(pre-surgical weight − reference weight obesity) [30]. To calculate reference weight obesity, the equation was used: [(height)^2^ × 25] [33].

Peripheral blood samples were collected in 5-mL Vacutainer™ tubes (BD) from the participants after they had fasted for at least 8 h. The following parameters were measured in peripheral blood samples: glucose, triglycerides, total cholesterol, high-density lipoprotein cholesterol (HDL-c), LDL-c, glycated hemoglobin (HbA1c), insulin, C-reactive protein (CRP), and the homeostatic model assessment of insulin resistance (HOMA-IR).

## Analysis of Lymphocyte Populations with Flow Cytometry

**Fig. 1:**
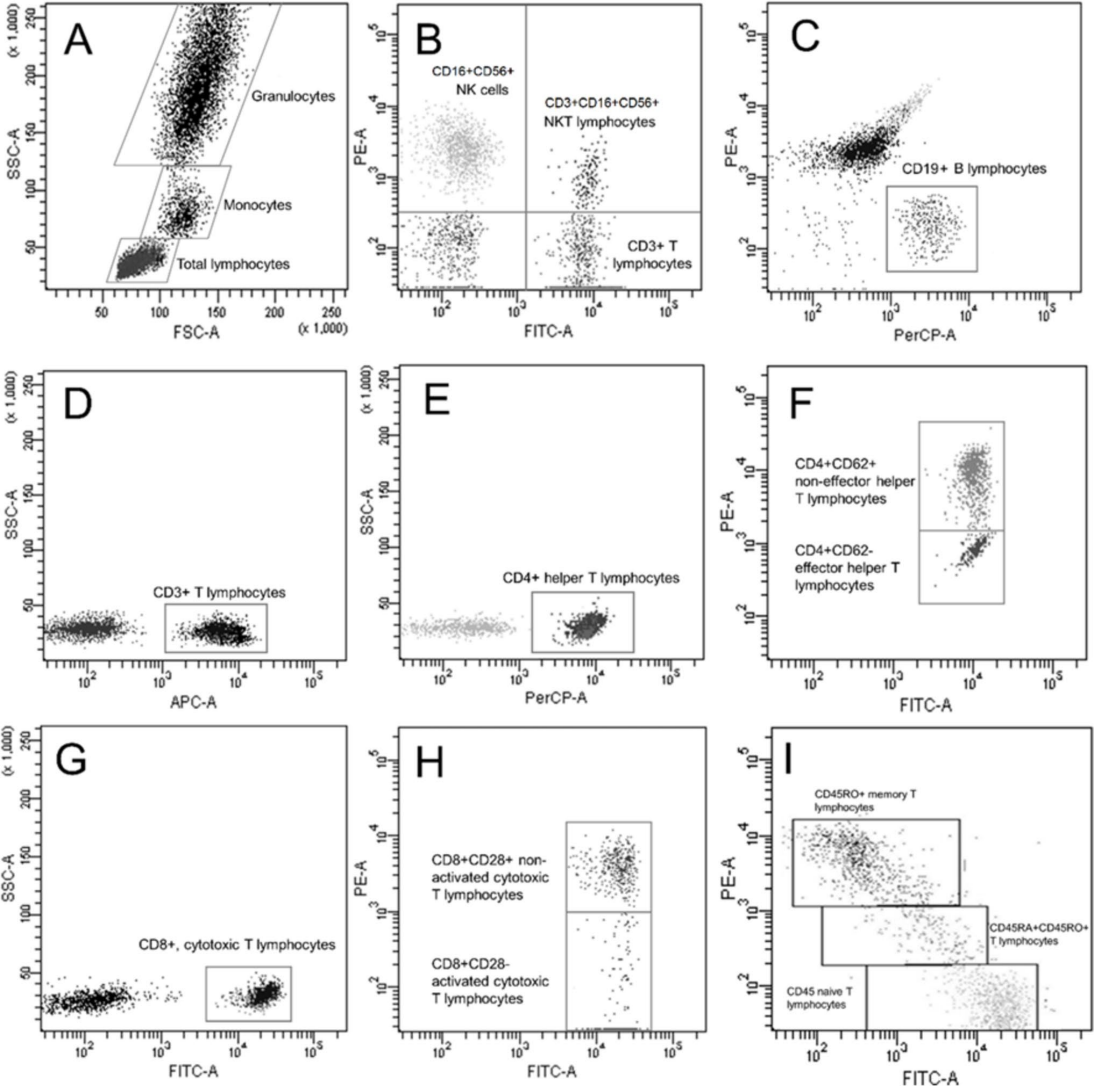
Immunofluorescence plots to determine the lymphocyte subpopulations with flow cytometry. **A** Control isotype with forward scatter (FSC)/side scatter (SSC) to identify lymphocytes, monocytes, and granulocytes. Note that FSC determined the cell size, and SSC determined the complexity of the cell. **B** and **C** T lymphocytes, natural killer (NK) cells, and B lymphocytes. **D**, **E**, and **G** Regionalization of T lymphocyte subpopulations (CD4+ and CD8+). **F** Activated T lymphocytes with helper functions. **H** Activated T lymphocytes with cytotoxic functions. **I** Naive helper and memory T lymphocytes and cytotoxic naive and memory T lymphocytes

Peripheral blood samples were collected in 5-mL Vacutainer™ tubes (BD). To identify the different cellular subpopulations, a mixture of commercial monoclonal antibodies conjugated to fluorochromes (BD) was employed: FITC-anti-CD3/PE-anti-(CD16+CD56)/PerCP-anti-CD19 (identifies T lymphocytes, NK cells, and B lymphocytes); FITC-anti-CD4/PE-anti-CD62L/APC-anti-CD3 (identifies activated T lymphocytes with helper functions); FITC-anti-CD8/PE-anti-CD28/APC-anti-CD3 (identifies activated T lymphocytes with cytotoxic functions); FITC-anti-CD45RA/PE-anti-CD45RO/PerCP-anti-CD4/APC-anti-CD3 (identifies naive and memory T lymphocytes); and FITC-anti-CD45RA/PE-anti-CD45RO/PerCP-anti-CD8/APC-anti-CD3 (identifies cytotoxic naive and memory T lymphocytes). The cells were incubated with the combination of antibodies. Subsequently, a lysis solution was added; the cells were washed in phosphate-buffered saline (PBS), and then fixed with 1% paraformaldehyde containing 0.1% sodium azide (NaN_3_). The samples were analyzed in a flow cytometer (FACScanto TM II, BD) within 24 h of staining. The analysis included a total of 10,000 cells for each event. Forward scatter and FL-3 scatter were employed to obtain the percentages of the desired cellular populations. Then, by-fluorescent dot plots were constructed to delimit the lymphocyte subpopulations [13, 34], utilizing the FACSDiva version 6.1.3 software (Fig. 1). The absolute number of each lymphocyte subpopulation was calculated as: (the percentage of the required lymphocyte subpopulation × the total number of lymphocytes)/100. The absolute values are expressed as cells/µL.

## Statistical Analysis

Data comparisons were determined based on cut-off points (1 month before and 6 months after surgery). The adjusted Kolmogorov–Smirnov method (Lilliefors test) was used to determine the normality of the data. The variables that did not pass the normality test were log transformed. The mean and standard deviation were calculated for the non-transformed variables, while the median and interquartile range were calculated for the transformed variables. The delta value (Δ) was calculated as the difference in a variable before surgery compared with after surgery. The percentage change after surgery was calculated for each variable by multiplying the mean delta (*M*Δ) by 100 and dividing it by the mean before surgery.

The paired *t*-test was applied to each variable to obtain the difference in the means and the *p*-value before and after surgery. One-way analysis of variance (ANOVA) was applied to each variable’s delta by sex to obtain the difference in the means and the *p*-value. Two-way ANOVA was applied to each variable’s delta by surgical technique, adjusted for sex, to obtain the difference in the means and the p-value. A Pearson correlation matrix was generated between the deltas of the metabolic and immune variables, considering associations with an *R* > 0.4. Statistical significance was set at *p* < 0.05 with a 95% confidence interval. The analysis was performed using statistical packages in the Python Environment v3.6.7 (CreateSpace 2009; Scotts Valley, CA, USA) and IBM SPSS Statistics version 25.0 (IBM Corp, Armonk, NY, USA).

## Results

We evaluated 50 individuals at two stages: 1 month before and 6 months after bariatric surgery. The individuals had a *mean* ± *standard deviation* age of 36.8 ± 9.4 years. Most of the patients were women (70%, *n* = 35). The LRYGB surgery group represented 60% (*n* = 30) of the patients, and the LSG group represented 40% (*n* = 20) of the patients. The follow-up rate was 88.7%. At the beginning of the study there were 67 individuals, 17 of whom were lost to follow-up: 6 of them dropped out of the study, 7 individuals were excluded due to post-surgical complications or due to consumption of any of the medications mentioned in the exclusion criteria, and 4 of the patients had missing data.

## Metabolic and Body Composition Changes

After LRYGB and LSG bariatric surgery, there is a decrease in body composition and metabolic parameters in the 50 participants, as shown in Table 1.

**Table 1:** Differences in the body composition and metabolic factors before and after bariatric surgery and by sex. The body composition and metabolic variables were compared for the entire study group 1 month before and 6 months after surgery, as outlined in “Materials and Methods”

Variable	Before surgery (*n* = 50)	After surgery (*n* = 50)	Percent change after surgery	*p*	*p*Δ@
* Weight* (kg)	115.0 ± 26.1	87.5 ± 19.8	↓ 24.1	** < 0.001**	** < 0.001**
*BMI* (kg/m^2^)	42.6 ± 7.0	32.3 ± 5.2	↓ 24.1%	** < 0.001**	**0.039**
*TBF* (%)	46.6 ± 6.9	35.9 ± 8.6	↓ 22.3%	** < 0.001**	0.997
*FFM* (%) (kg)	53.3 ± 6.9	64.0 ± 8.6	↑19.1%	** < 0.001 < 0.001**	0.997 **0.012**
	54.3 (50.0–75.7)	49.5 (45.6–66.7)	↓ 9.7%		
*WC* (cm)	127.6 ± 17.8	107.9 ± 15.9	↓ 16.1%	** < 0.001**	0.194
*VF* (U)	18.5 (13–20)	8 (7–12)	↓ 43.4%	** < 0.001**	**0.042**
Glucose (mg/dL)	104.0 (95.4–118.6)	87.0 (80.0–93.5)	↓ 22.8%	** < 0.001**	0.354
Insulin (µUI/mL)	22.5 (15.0–31.6)	7.9 (5.7–12.0)	↓ 56.2%	** < 0.001**	0.205
HbA1c (%)	5.7 (5.5–6.0)	5.3 (5.0–5.4)	↓ 15.6%	** < 0.001**	0.145
HOMA-IR	5.9 (3.8–9.3)	1.5 (1.1–2.5)	↓ 66.0%	** < 0.001**	0.127
Total cholesterol (mg/dL)	177.6 ± 31.4	153.1 ± 23.0	↓ 12.9%	** < 0.001**	0.639
HDL-c (mg/dL)	40.4 (35.0–47.2)	43.0 (36.0–49.5)	↑ 2.4%	0.075	0.989
LDL-c (mg/dL)	102.5 ± 27.2	88.2 ± 18.9	↓ 13.1%	**0.001**	0.649
Triglycerides (mg/dL)	150.0 (106.7–195.0)	110.0 (86.5–136.0)	↓ 30.5%	** < 0.001**	0.822
*CRP* (mg/dL)	0.767 (0.315–1.055)	0.234 (0.104–0.630)	↓ 49.2%	** < 0.001**	0.802
*SBP* (mmHg)	121.3 ± 11.7	110.9 ± 10.8	↓ 9.1%	** < 0.001**	0.616
*DBP* (mmHg)	80 (70–84)	70 (69–77.5)	↓ 7.9%	** < 0.001**	0.376

*p* statistical significance before versus after bariatric surgery, *p*Δ@ statistical significance by sex (mean delta). *p* < 0.05 is statistically significant. In the percentage change column, the up arrow (↑) indicates an increase, and the down arrow (↓) indicates a decrease. The data are presented as the *mean* ± *standard deviation* or median (interquartile range)

*BMI* body mass index, *WC* waist circumference, *TBF* total body fat, *FMM* free muscle mass, *VF* visceral fat, *HbA1c* glycated hemoglobin, *HOMA-IR* homeostatic model assessment of insulin resistance, *HDL-c* high-density cholesterol, *LDL*-c low-density cholesterol, *CRP* C-reactive protein, *SBP* systolic blood pressure, *DBP* diastolic blood pressure, *n* number of individuals

## Changes in Lymphocyte Subpopulations

**Fig. 2:**
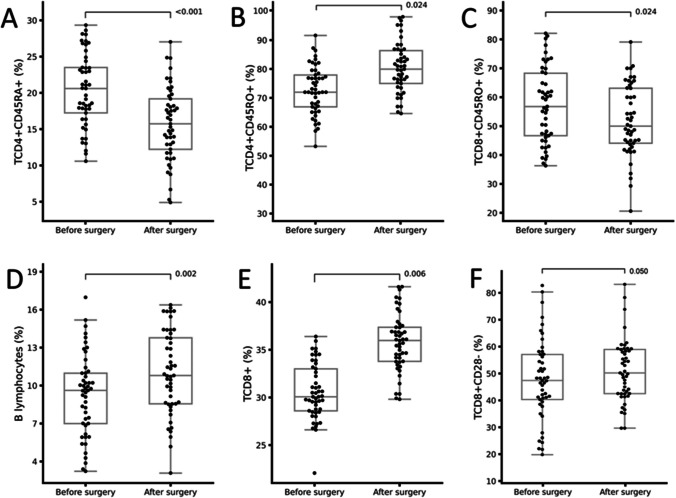
Featured differences in immune cells before and after bariatric surgery in the study group **A** Naive helper T lymphocytes, **B** memory helper T lymphocytes, **C** memory cytotoxic T lymphocytes, **D** B lymphocytes, **E** cytotoxic T lymphocytes, and **F** effector cytotoxic T lymphocytes. *p* < 0.05 indicates a statistically significant difference

In addition to changes in body composition and metabolic factors, there were differences in B lymphocytes (Fig. 2D, p < 0.002), CD4 + CD45RO + memory T lymphocytes (Fig. 2B, p < 0.022), CD8+ T lymphocytes (Fig. 2E, p < 0.002), and CD8+CD28− effector T lymphocytes (Fig. 2F, p < 0.000), which increased after bariatric surgery. In contrast, CD4+CD45RA+ naive T lymphocytes (Fig. 2A, p < 0.001) and CD8+CD45RO+ memory T lymphocytes (Fig. 2C, p < 0.03) decreased after the intervention (Table 2, Fig. 2).

**Table 2:** Differences in lymphocyte subpopulations. The relative (%) and absolute (cells/µL) numbers were compared by sex 1 month before and 6 months after surgery

Variable	Before surgery (*n* = 50)	After surgery (n = 50)	*p*	*p*Δ@
Leukocytes	7615 (6525–9725)	6000 (5300–7850)	** < 0.001**	0.960
Monocytes	8.0 (6.0–9.2)	7.0 (5.1–9.3)	0.820	0.537
	617.4 (456.3–794.3)	475 (333.1–599.2)	**0.001**	0.552
Granulocytes	64.9 ± 10.6	64.3 ± 10.2	0.812	0.576
	5463.9 ± 2073.8	4255.7 ± 1413.8	** < 0.001**	0.909
Total lymphocytes	27.1 ± 10.2	27.0 ± 9.7	0.807	0.713
	2223.6 ± 979.4	1796.4 ± 719.1	0.050	0.843
CD16+CD56+ natural killer cells	18.8 (12.5–25.4)	16.6 (8.9–22.9)	0.122	**0.049**
	346.4 (232.2–523.9)	279.6 (153.0–246.9)	**0.001**	0.162
CD3+CD16+CD56+ TNK lymphocytes	3.3 (2.4–5.6)	3.6 (2.2–3.6)	0.905	0.908
	70.4 (41–93.5)	65.4 (38.5–86.1)	0.604	0.820
CD19+ B lymphocytes	9.4 (6.2–11.6)	11.3 (8.0–18.3)	**0.002**	0.380
	167.1 (109.6–322.3)	184.3 (124.3–212.1)	0.580	0.273
CD3+ T lymphocytes	67.0 ± 11.2	64.5 ± 10.5	0.225	0.232
	1506.2 ± 707.3	1171.8 ± 538.9	** < 0.001**	0.413
CD4+ helper T lymphocytes	60.2 (51.2–64.9)	55.5 (49.5–64.7)	0.272	0.299
	1186.1 (902.0–1658.5)	859 (668.8–1299.2)	** < 0.001**	0.870
CD4+CD62− effector helper T lymphocytes	63.4 ± 11.8	65.5 ± 15.2	0.443	0.966
	1274.6 (870.3–2018.6)	1165.1 (773.5–1641.9)	**0.019**	0.656
CD4+CD62+ non-effector helper T lymphocytes	36.9 ± 11.9	33.4 ± 15.7	0.367	0.951
	700.8 (503.7–965.7)	522.7 (291.1–929.1)	**0.002**	0.949
CD4+CD45RA + naïve helper T lymphocytes	22.9 (16.4–30.5)	15.2 (10.7–21.2)	** < 0.001**	0.684
	474.5 (268.9–633.5)	266.6 (186.6–350.2)	** < 0.001**	0.496
CD4+CD45RO+ memory helper T lymphocytes	67.2 (60.3–73.2)	73.0 (66.7–79.4)	**0.022**	0.387
	1388.6 (899.2–1733.4)	1309.8 (866.4–1687.8)	0.283	0.370
CD4+CD45RA+CD45RO+ double-positive helper T lymphocytes	10 (7.4–13.7)	8.4(6.3–11.8)	**0.030**	0.423
	207.7 (150.6–278.1)	143.6 (93.7–221.5)	** < 0.001**	0.303
CD8+ cytotoxic T lymphocytes	30 (26.1–39.4)	36.7 (29.9–42.0)	**0.006**	0.549
	637.3 (435.8–896.2)	621.0 (436.1–870.4)	0.304	0.575
CD8+CD28− effector cytotoxic T lymphocytes	59.9 ± 16.9	61.0 ± 14.7	**0.050**	0.849
	1240.9 ± 704.2	1065.3 ± 435.7	**0.049**	0.933
CD8+CD28+ non-effector cytotoxic T lymphocytes	44.5 ± 16.6	38.7 ± 14.7	0.066	0.998
	844.4 (577.4–1246.0)	598.6 (384.7–867.4)	**0.001**	0.887
CD8+CD45RA+ naive cytotoxic T lymphocytes	27.3 ± 9.0	31.4 ± 9.4	0.057	0.585
	600 (374.4–729.8)	506.7 (376.0–712.6)	0.283	0.590
CD8+CD45RO+ memory cytotoxic T lymphocytes	57.5 ± 12.9	51.8 ± 12.2	**0.024**	0.836
	1292.9 (830.4–1637.9)	940.7 (561.3–1196.2)	** < 0.001**	0.726
CD8+CD45RA+CD45RO+ double-positive cytotoxic T lymphocytes	14.5 ± 6.4	16.1 ± 6.2	0.246	0.670
	281.2 (176.6–396.8)	288.9 (173.2–428.2)	0.566	0.706

*p* statistical significance before versus after bariatric surgery; *p*Δ@ statistical significance by sex. *p* < 0.05 is statistically significant. The data are presented as the *mean* ± *standard deviation* or median (interquartile range)

Using the aforementioned results, we performed a regression analysis to search for correlations between the percentage of lymphocyte subpopulations, body composition variables, and biochemical variables; not finding significant correlations.

## Differences in Body Composition and Metabolism Between Patients Who Underwent LRYGB and LSG

Table 3 shows the comparison of body composition and metabolic variables between the two LRYGB and LSG groups. The patients who underwent LRYGB showed improvements in all metabolic and body composition markers, except for HDL-c. The patients who underwent LSG also showed significant metabolic improvements, except for total cholesterol, HDL-c, and LDL-c.

**Table 3:** Differences in body composition and metabolism between the bariatric surgical techniques. Body composition and the metabolic variables were compared by sex between patients who underwent LRYGB and patients who underwent LSG 1 month before and 6 months after surgery

Variable (*n* = 50)	LRYGB			LSG			LRYGB versus LSG	
	*n* = 30		*p*	*n* = 20		*p*	*p*Δ	*p*Δ@
	Before	After		Before	After			
Weight (kg)	120 ± 24	89 ± 18	** < 0.001**	108 ± 28	85 ± 22	** < 0.001**	**0.002**	** < 0.001**
BMI (kg/m^2^)	44 ± 6.8	33 ± 5	** < 0.001**	41 ± 7	32 ± 6	** < 0.001**	**0.002**	**0.003**
TBF (%)	46 ± 6.7	35 ± 10	** < 0.001**	48 ± 7	39 ± 6	** < 0.001**	**0.002**	0.467
FFM (%) (kg)	54 ± 6.7	65 ± 10	** < 0.001** ** < 0.001**	53 ± 7	61 ± 6	** < 0.001** **0.020**	0.197 0.190	0.438 **0.043**
	58 (52–78)	50 (46–70)		52 (47–60)	48 (46–53)			
WC (cm)	132 ± 17	111 ± 17	** < 0.001**	122 ± 17	101 ± 13	** < 0.001**	0.965	0.482
VF (U)	19 (13–21)	9 (7–13)	** < 0.001**	18.0 (11–20)	8 (7–10)	** < 0.001**	**0.037**	**0.024**
Glucose (mg/dL)	111 (98–126)	90 (83–95)	** < 0.001**	101 (93–106)	86 (78–91)	** < 0.001**	0.414	0.347
Insulin (µUI/mL)	23 (16–34)	8 (6–12)	** < 0.001**	21 (13–31)	9 (6–14)	**0.001**	0.155	0.361
HbA1c (%)	5.9 (5.6–6.9)	5.1 (4.9–5.5)	** < 0.001**	5.6 (5.5–5.8)	5.3 (5.1–5.4)	** < 0.001**	**0.031**	0.082
HOMA-IR	6.3 (4.3–10.4)	1.5 (1.2–2.5)	** < 0.001**	5.6 (2.9–8.5)	1.6 (1.1–2.8)	** < 0.001**	0.251	0.211
Total cholesterol (mg/dL)	180 ± 33	145 ± 25	** < 0.001**	175 ± 30	169 ± 16	0.451	0.974	0.976
HDL-c (mg/dL)	40 (34–44)	43 (36–48)	0.108	41 (37–52)	45 (38–56)	0.397	0.991	1.000
LDL-c (mg/dL)	103 ± 27	81 ± 19	** < 0.001**	104 ± 22	101 ± 13	0.655	**0.022**	**0.047**
Triglycerides (mg/dL)	164 (98–208)	119 (111–136)	**0.001**	112 (89–116)	115 (83–136)	**0.001**	0.985	**0.015**
CRP (mg/dL)	0.825 (0.235–1.160)	0.237 (0.153–0.818)	** < 0.001**	0.570 (0.238–1.035)	0.210 (0.104–0.547)	**0.024**	0.752	0.939
SBP (mmHg)	124 ± 12	112 ± 10	** < 0.001**	120 ± 14	109 ± 11	**0.015**	0.810	0.784
DBP (mmHg)	80 (70–84)	71 (70–80)	**0.001**	78 (69–80)	70 (66–76)	**0.010**	0.331	0.461

*p* statistical significance before versus after bariatric surgery; *p*Δ statistical significance between LRYGB and LSG; *p*Δ@ statistical significance between LRYGB and LSG (adjusted for sex). *p* < 0.05 is statistically significant. The data are presented as the *mean* ± *standard deviation* or the median (interquartile range)

*LRYGB* laparoscopic Roux-en-Y gastric bypass, *LSG* laparoscopic sleeve gastrectomy, *BMI* body mass index, *WC* waist circumference, *TBF* total body fat, *FMM* free muscle mass, *VF* visceral fat, *HbA1c* glycated hemoglobin, *HOMA-IR* homeostatic model assessment of insulin resistance, *HDL-c* high-density cholesterol, *LDL-c* low-density cholesterol, *CRP* C-reactive protein, *SBP* systolic blood pressure, *DBP* diastolic blood pressure, *n* number of individuals

**Fig. 3:**
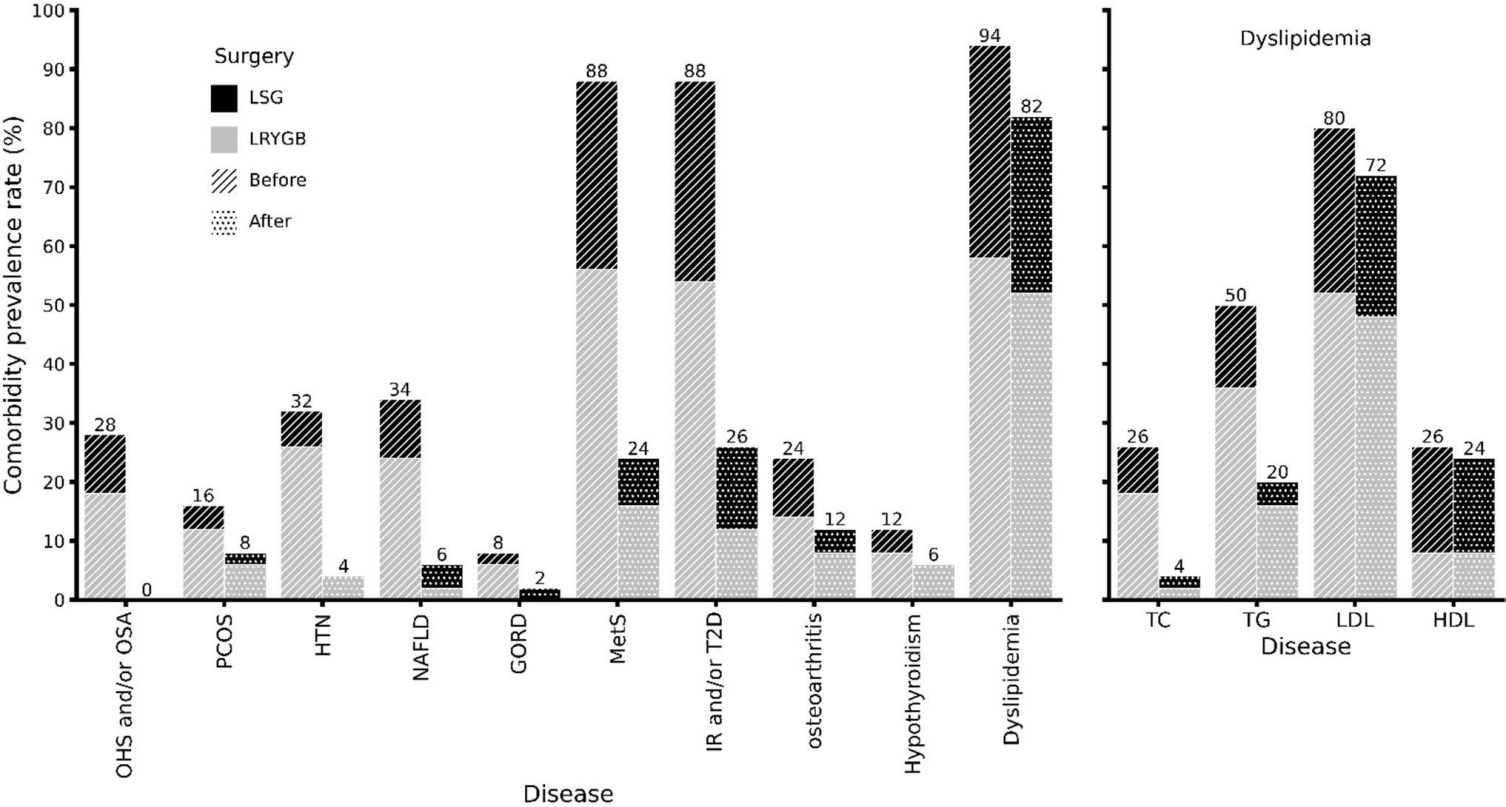
The prevalence of obesity-associated medical problems before and after the bariatric surgical procedure. Abbreviations: — *LSG* laparoscopic sleeve gastrectomy, *LRYGB* laparoscopic Roux-en-Y gastric bypass, *OHS* obesity hypoventilation syndrome, *OSA* obstructive sleep apnea, *PCOS* polycystic ovary syndrome, *HTN* hypertension, *NALF* nonalcoholic fatty liver disease, *GORD* gastroesophageal reflux, *MetS* metabolic syndrome, *IR* insulin resistance, *T2D* type 2 diabetes, *TC* hypercholesterolemia, *TG* hypertriglyceridemia, *LDL* low-density lipoprotein hypercholesterolemia, and *HDL* high-density lipoprotein hypoalphalipoproteinemia

The analysis of the clinical or medical records of each patient revealed there was a decrease in the prevalence of obesity-associated medical problems in the patients after both bariatric surgical procedures. In general, LRYGB promoted improvement of obesity-associated medical problems and metabolic clinical signs compared with LSG (Fig. 3).

The analysis of the clinical or medical records of each patient revealed there was a decrease in the prevalence of obesity-associated medical problems in the patients after both bariatric surgical procedures. In general, LRYGB showed a greater reduction in FFM (mainly in women) measured in kilograms and promoted improvement of obesity-associated medical problems and metabolic clinical signs, compared with LSG (Fig. 3).

## Differences in Lymphocyte Subpopulations Between Patients Who Underwent LRYGB and LSG

Patients who underwent LRYGB showed a decrease in the percentage of monocytes and CD4+ T lymphocytes, including naive (CD4+CD45RA+), double-positive (CD4+CD45RA+CD45RO+), and memory (CD8+CD45RO+) T lymphocytes. By contrast, B lymphocytes, CD8+ T lymphocytes, and CD4+CD45RO+ T lymphocytes increased after surgery. In patients who underwent LSG, there were changes only in CD4+CD62- and CD4+CD45RA+ T lymphocytes. Comparing the two surgical techniques, LSG promoted a greater decrease in CD4+CD62- T lymphocytes than LRYGB (Table 4).

**Table 4:** Differences in the lymphocyte subpopulations between patients who underwent LRYGB and LSG. Relative (%) and absolute (cells/µL) numbers were compared by surgical technique and sex 1 month before and 6 months after surgery

Variable (*n* = 50)	LRYGB			LSG			LRYGB versus LSG	
	*n* = 30		*p*	*n* = 20		*p*	*p*Δ	*p*Δ@
	Before	After		Before	After			
Leukocytes	8700 (6600–11225)	6500 (5700–7900)	** < 0.001**	7730 (6250–9100)	5800 (4800–7550)	**0.009**	0.203	0.443
Monocytes	8 (6–9)	7 (5–9)	**0.488**	8 (6–10)	7 (5–10)	0.216	0.201	0.100
	617 (454–814)	475 (383–599)	**0.020**	648 (458–816)	400 (261–621)	**0.018**	0.955	0.879
Granulocytes	64 ± 12	62.8 ± 10.2	0.678	66 ± 9	67 ± 10	0.868	0.691	0.751
	5674 ± 2310	4354 ± 1260	** < 0.001**	5174 ± 1626	4251 ± 1645	0.055	0.276	0.527
Total lymphocytes	29 ± 10	29 ± 11	0.784	25 ± 9	28 ± 15	0.967	0.510	0.182
	2429 ± 1025	1942 ± 756	**0.002**	1919 ± 817	1628 ± 635	0.088	0.316	0.605
CD16 + CD56 + natural killers cells	19 (12–25)	17 (9–22)	0.205	19 (14–26)	15 (9–29)	0.391	0.456	0.109
	375 (259–653)	279 (165–474)	**0.008**	303 (209–475)	258 (131–387)	0.062	0.605	0.229
CD3+CD16+ CD56 + TNK lymphocytes	3 (2–6)	4 (2–5)	0.928	4 (2–7)	4 (2–7)	0.659	0.909	0.984
	65 (41–81)	65 (43–80)	0.788	83 (30–109)	67 (24–104)	0.182	0.365	0.612
CD19+ B lymphocytes	9 (6–11)	11 (8–19)	**0.004**	10 (7–15)	12 (10–17)	0.160	0.274	0.297
	170 (135–325)	176 (132–325)	0.538	164 (102–332)	187 (118–287)	0.916	0.321	0.245
CD3+ T lymphocytes	68 ± 11	66 ± 11	0.360	65 ± 11	63 ± 10	0.430	0.893	0.491
	1665 ± 733	1296 ± 596	**0.003**	1259 ± 593	990 ± 389	0.069	0.518	0.634
CD4+ helper T lymphocytes	60 (53–65)	55 (49–63)	**0.041**	61 (51–66)	57 (50–69)	0.549	0.083	0.080
	1294 (936–1798)	822 (694–1390)	**0.001**	1037 (693–1423)	884 (587–1239)	0.223	0.105	0.242
CD4+CD62− effector helper T lymphocytes	35 ± 11	37 ± 16	0.422	38 ± 14	28 ± 14	**0.039**	**0.023**	0.070
	760 (540–982)	745 (337–1054)	**0.043**	592 (431–978)	350 (245–578)	**0.026**	0.594	0.744
CD4+CD62+ non-effector helper T lymphocytes	64 ± 11	62 ± 16	0.440	61 ± 14	70 ± 13	0.054	0.699	0.703
	1325 (931–2196)	1166 (733–1735)	**0.005**	989 (708–1713)	1047 (767–1458)	0.728	0.153	0.355
CD4+CD45RA+ naive helper T lymphocytes	24 (16–33)	15 (11–19)	**0.001**	20 (17–29)	18 (10–24)	**0.049**	0.551	0.729
	547 (317–699)	266 (192–333)	** < 0.001**	382 (241–538)	265 (144–378)	**0.018**	0.486	0.520
CD4+CD45RO+ memory helper T lymphocytes	67 (57–73)	76 (72–81)	0.050	67 (61–74)	68 (66–76)	0.383	0.140	0.168
	1430 (1044–1951)	1460 (986–1771)	0.445	1150 (785–1504)	1202 (658–1359)	0.466	0.908	0.644
CD4+CD45RA+CD45RO+ double-positive helper T lymphocytes	10 (8.0–15.2)	8 (6–12)	**0.010**	11 (7–13)	9 (7–13)	0.874	0.170	0.215
	228 (153–326)	143 (97–203)	** < 0.001**	204 (122–264)	143 (80–231)	0.249	0.471	0.160
CD8+ Cytotoxic T lymphocytes	30 (27–38)	37 (31–46)	**0.004**	31 (26–42)	34 (28–40)	0.377	0.674	0.396
	689 (555–1020)	802 (466–1084)	0.551	521 (373–724)	501 (427–673)	0.404	0.352	0.603
CD8+CD28− effector cytotoxic T lymphocytes	45 ± 17	39 ± 16	0.270	45 ± 18	38 ± 14	0.136	0.603	0.871
	873 (645–1266)	598 (446–1123)	**0.006**	636 (445–1199)	605 (284–696)	**0.044**	0.829	0.969
CD8+CD28+ non-effector cytotoxic T lymphocytes	56 ± 16	60 ± 16	0.262	54 ± 17	62 ± 14	0.090	0.533	0.819
	1400 ± 747	1155 ± 504	**0.042**	1002 ± 557	934 ± 377	0.620	0.288	0.538
CD8+CD45RA+ naive cytotoxic T lymphocytes	27 ± 10	32 ± 10	0.061	28 ± 8	31 ± 8	0.305	0.450	0.589
	633 (370–821)	515 (376–845)	0.390	545 (377–653)	489 (373–570)	0.538	0.834	0.861
CD8+CD45RO+ memory cytotoxic T lymphocytes	58 ± 14	51 ± 13	**0.031**	56 ± 12	53 ± 12	0.350	0.929	0.686
	1314 (839–1809)	937 (616–1205)	** < 0.001**	1199 (753–1399)	946 (540–1132)	0.091	0.299	0.501
CD8+CD45RA+CD45RO+ double positive cytotoxic T lymphocytes	14 ± 6	16 ± 7	0.286	15 ± 7	16 ± 5	0.605	0.801	0.900
	287 (169–470)	288 (178–476)	0.441	290 (184–370)	290 (163–333)	0.954	0.627	0.794

*p* statistical significance before versus after bariatric surgery; *p*Δ statistical significance between LRYGB and LSG; *p*Δ@ statistical significance between LRYGB and LSG adjusted for sex. *p* < 0.05 is statistically significant. The data are presented as the *mean* ± *standard deviation* or median (interquartile range)

*LRYGB* laparoscopic Roux-en-Y gastric bypass, *LSG* laparoscopic sleeve gastrectomy

## Discussion

After undergoing bariatric surgery, patients with severe obesity showed significant metabolic and clinical improvements, except for HDL-c cholesterol. HDL-c is considered a pro-inflammatory biochemical marker and tends to decrease in the presence of inflammation [35]. In the study group, HDL-c remained decreased after surgery. It is known that in the Latin population, HDL-c levels are lower due to genetic, sociocultural, and environmental factors (low physical activity) compared with other populations [36]. It is possible that the minor improvement in HDL-c could also be related to a small sample size, compared to other studies with large samples that showed a major decrease in HDL levels [37–39]. It may also be because LSG and LRYGB are surgical techniques with less metabolic power compared with biliopancreatic diversion with duodenal switch (BPD-DS) [40, 41] and one anastomosis gastric bypass (OAGB) [42–44].

They achieved normal glucose, insulin, HbA1c, HOMA-IR, total cholesterol, triglycerides, CRP, and DBP at just 6 months after treatment. These findings confirm the metabolic benefits of bariatric surgical procedures and a multidisciplinary team (including bariatric specialists, nutritionists, and psychologists), contributing to the reduction of morbidity and mortality from metabolic diseases [45–48].

Despite the notable improvements, the patients still had percentage of % EWL relative to their ideal or healthy weight, and the mean BMI, TBF, and WC were high. According to the American Society of Metabolic and Bariatric Surgery (ASMBS) and The Obesity Society, American Society for Metabolic and Bariatric Surgery, Obesity Medicine Association, and American Society of Anesthesiologists [49, 50], individuals undergoing LRYGB are expected to have an EWL percentage greater than 60% at 1 year after bariatric surgery, which is considered an excellent weight loss outcome. In the present study, patients undergoing LRYGB and LSG achieved an EWL percentage of 64.8% and 57.6%, respectively, as early as 6 months after surgery.

Women who underwent LRYGB showed a greater reduction in FFM measured in kilograms, compared with women who underwent LSG (Table 3). As in other studies [51, 52], FFM loss may vary depending on the surgical procedure, the degree of weight loss, the frequency and intensity of physical activity, dietary protein intake, and the assessment method used [52, 53].

It must be admitted that the present study has limitations. The sample size is small, which increases the heterogeneity of the cohort when dividing it to compare the two surgical techniques by sex (mostly women undergoing LRYGB). Sex is a factor that influences obesity, as well as participation in studies and health programs. Having explored immunometabolic results only up to 6 months of follow-up may be a contributing factor since weight loss is not usually at its lowest point, so improvements in medical problems associated with obesity should be interpreted with caution. Therefore, we consider in the future to do a more complete sampling and a longer follow-up (at least for 12 months) to have a more balanced population.

## Changes in T Lymphocyte Subpopulations and Their Relationship with Metabolic Improvements After Bariatric Surgery

Obesity has been identified as a factor that alters the delicate balance of immune cells in AT toward a pro-inflammatory state, accounting in part for the systemic effects of chronic inflammation [18, 20, 54, 55]. In the present study, there was a decrease in some lymphocyte subpopulations in the blood, consistent with other studies in adult populations that have undergone bariatric surgery [31, 47, 56].

In the present study, we observed an increase in the percentage of memory helper T lymphocytes (CD4+CD45RO+) after bariatric surgery. These cells have a long lifespan and express large amounts of cytokines and anti-apoptotic proteins, including interleukin 77 (IL-77) and IL-15, which block programmed cell death [57]. In aging and obesity, there is an increase in the proportion of CD4+CD45RO+ T lymphocytes, contributing to the generation and perpetuation of chronic inflammation [58, 59]. Therefore, the decrease in obesity due to surgery would be expected to decrease this subpopulation. Rizk et al. [60] reported a decrease in the percentage of effector memory T lymphocytes, but there was an increase in central memory T lymphocytes after LSG. In the present study, the patients still had obesity 6 months after surgery, despite achieving a relative medium–low BMI and therefore some degree of inflammation, which is probably the reason why the proportion of these cells still showed an alteration.

We also found a decrease in the percentage of naive helper T lymphocytes (CD4+CD45RA+ ; Tables 2 and 4). Similar studies have reported a positive association between a reduction in the percentage of total helper T cells and weight loss and *BMI* [61, 62]. Naive T lymphocytes are mature cells that have differentiated and successfully passed the selection processes in the thymus, but they have not yet encountered their specific antigen in the periphery [10]. Similarly, Jongbloed et al. [63] reported a decrease in this T lymphocyte subpopulation after bariatric surgery. The decrease in naïve CD4+T lymphocytes in bariatric patients could be associated with decreased calorie intake and weight loss, which reduces overstimulation of the interleukin 7 receptor (IL-7R) and therefore levels of IL-7, a cytokine that in animal models has been shown to control the homeostasis of these cells [64, 65].

We observed an increase in the percentage of CD8+ T lymphocytes (Tables 2 and 4 and Fig. 2E) and CD8+CD28 − effector T lymphocytes (Table 2 and Fig. 2F), mainly in patients who underwent LRYGB (Table 4). These cytotoxic T lymphocytes have a high capacity to react to pathogens and to differentiate into effector cells that migrate to sites of damage to eliminate infected and/or cancerous cells [61, 63, 66, 67]. We observed an increase in the percentage of CD8 + T lymphocytes (Tables 2 and 4 and Fig. 2E) and CD8+CD28− effector T lymphocytes (Table 2 and Fig. 2F), mainly in patients who underwent LRYGB (Table 4). In individuals with obesity, excessive activation of cytotoxic T lymphocytes contributes to the generation and perpetuation of inflammation [19, 58, 59]. Therefore, the decrease in these cells after surgery could indicate an improvement in obesity-related inflammation [58]. Although there were improvements in the metabolic and clinical parameters, the presence of these cells in our patients suggests that the inflammatory process has not completely disappeared. Therefore, we consider it important to conduct additional studies on CD8+ T lymphocytes and their phenotypic variables in similar populations to clarify these discrepancies.

We noted a decrease in the total number of CD4+ helper T lymphocytes, mainly in individuals who underwent LRYGB (Table 4). This finding is consistent with other studies that have also reported a decrease in CD4+ T lymphocytes [60]. Additionally, this reduction has been associated with weight loss, BMI [61, 62], VF, HbA1c [68], and insulin resistance [63, 67]. This suggests that the reduction in immune activity mediated by these cells restricts low-grade inflammation associated with obesity [59].

## Change in the Percentage of B Lymphocytes After Bariatric Surgery

As the severity of obesity increases, so does the number of B lymphocytes, as they are among the first immune cells to infiltrate visceral adipose tissue (VAT) and then the bloodstream [16, 69, 70]. This increase has also been implicated in glucose metabolism disorders associated with obesity [15] and in the risk of autoimmune diseases [71].

In this study, there was an increase in B lymphocytes after bariatric surgery (Fig. 2D), mainly in patients who underwent LRYGB (Table 3). This finding is comparable to another study in which the authors observed an increase in the percentage of memory B lymphocytes [72]. Furthermore, after surgery, B lymphocytes adjusted to the percentages found in patients with normal weight, although the authors concluded that the reduction in the proportion of these cells is not related to their functionality [73]. B lymphocytes produce immunoglobulins (IGs), which are an important part of the body’s defense [10]. Tobón et al. [74] did not observe changes in the percentages of B lymphocytes after bariatric surgery. They demonstrated that the decrease in IGs, mainly IgG and autoimmune IgM (anticardiolipin) was not related to the proportion of these cells in the blood. In contrast, Ballesteros-Pomar et al. [67] observed a decrease in B lymphocytes in the blood after surgery, but if these data are converted to total numbers, then the results correspond with the literature. Furthermore, Cuellar-Tamez et al. [75] noted that after surgery there was a decrease in the plasma levels of IgG and molecules that promote survival and B lymphocyte isotype change, which correlated with a decrease in their activity, glucose, and HOMA-IR. These changes have been attributed to weight loss because caloric deprivation and nutritional deficiencies induced by bariatric surgery can lead, to some extent, to immunodeficiency and the development of autoimmune diseases.

Modulation of pro-inflammatory T lymphocytes and an improvement in the function of effector B lymphocytes (which change from an effector to a regulatory phenotype) after bariatric surgery lead to an increase in the production of IL-10 and transforming growth factor beta (TGF-β), as well as a decrease in the secretion of interferon gamma (INF-γ), IL-2, IL-4, and IL-17 [76], leading to an increase in regulatory B lymphocytes [72]. Furthermore, in a previous study we observed that as the degree of obesity increases, the percentage of peripheral B lymphocytes decreases [77]. In the present study, there was an increase in these cells in the periphery, which could be sign of recovery and a decrease in chronic inflammation in the patients. Based on our results, we formulated two hypotheses: (1) The increase in B lymphocytes is associated with a shift toward a regulatory phenotype and (2) The increase in B lymphocytes is a sign of recovery and a decrease in chronic inflammation in these patients who have lost significant weight. However, testing these hypotheses requires new studies in populations with similar characteristics, which determine cellular function by incorporating molecules such as cytokines and antibodies as well as different phenotypic variants in peripheral blood and AT.

## There Are Immunometabolic Changes Between Gastric Bypass and Sleeve Gastrectomy

According to our data and patients’ clinical reports, those who underwent LRYGB showed greater metabolic disease improvement (Table 3, Fig. 3), as well as more significant changes in lymphocyte subpopulations (Table 4). These findings are consistent with other studies [62, 66, 78]. But curiously, sleeve gastrectomy produces a greater decrease in effector T helper lymphocytes. This can be considered beneficial as results in a decrease in the production of pro-inflammatory cytokines and directly influences obesity-associated inflammation, especially systemic inflammation that causes numerous comorbidities [79].

## Conclusion

Bariatric surgery, mainly LRYGB, leads to greater immunometabolic changes probably because it reduces inflammation and improves associated metabolic problems. After bariatric surgery, all metabolic variables showed significant improvements except HDL-c, perhaps due to ethnic factors, prevailing inflammation and small sample size. After surgery, a reduction in the count naive T helper and memory cytotoxic T lymphocytes stands out; these can be considered in the future as more precise clinical markers that help detect the decrease in inflammation related to obesity. Additionally, there was an increase in B, memory helper T, and cytotoxic T lymphocytes; these latter cells are probably related to the inflammatory process that persists after surgery, despite reaching a relative medium–low BMI. Therefore, patients with a higher degree of obesity and the presence of associated medical problems could benefit more from LRYGB, if we ensure that there is not excessive loss fat-free mass (particularly in women) by promoting physical activity and ensuring minimum protein consumption in the diet; this could help in the surgeon’s decision to select the surgical technique. It is important to continue to conduct studies in similar populations to determine the phenotypic variations of B, memory helper T, and cytotoxic T lymphocytes, as well as their pro-inflammatory and anti-inflammatory activity in both blood and adipose tissue, as well as similar studies in a longer-term and with greater metabolic efficiency techniques, like BPD-DS, OAGB, or single anastomosis gastro-ileal bypass (SAGI), to offer results with greater evidence.
